# Cervical Dysplasia and Cervical Cancer During Pregnancy: From Pathogenesis to Clinical Management

**DOI:** 10.3390/jcm14113784

**Published:** 2025-05-28

**Authors:** Aleksandra Piórecka, Weronika Marcinkowska, Filip Gągorowski, Magdalena Gąsior, Katarzyna Kazimierczuk, Agnieszka Żalińska, Przemysław Oszukowski, Agnieszka Pięta-Dolińska

**Affiliations:** 1 Department of Obstetrics and Perinatology, Medical University of Lodz, 90-419 Lodz, Poland; aaleksandra.nowakowska@gmail.com (A.P.); a.b.zalinska@gmail.com (A.Ż.); oszukowskip@gmail.com (P.O.); pietadol@gmail.com (A.P.-D.); 2 Labour Department, Polish Mother’s Memorial Hospital Research Institute, 93-338 Lodz, Poland; 3 Faculty of Medicine, Medical University of Lodz, 90-419 Lodz, Poland; filip.g.fgg@gmail.com (F.G.); magdalena.gasior@stud.umed.lodz.pl (M.G.); katarzyna.kazimierczuk@stud.umed.lodz.pl (K.K.); 4 Department of Gynecology, Reproduction and Fetal Therapy and Infertility Diagnostics and Treatment, Polish Mother’s Memorial Hospital Research Institute, 93-338 Lodz, Poland

**Keywords:** cervical dysplasia, cervical cancer, human papillomavirus

## Abstract

The incidence of malignancies diagnosed during pregnancy is estimated at 1 in 1000 pregnancies, with cervical cancer being the most common gynecological malignancy in this population. The increasing maternal age and widespread use of prenatal screening contribute to the rising detection rates. Early symptoms of cervical cancer, such as vaginal bleeding or discharge, often mimic normal pregnancy changes, leading to potential delays in diagnosis. Cervical dysplasia, a known precursor of cervical cancer, is closely associated with high-risk HPV infection, which affects approximately 25% of women of reproductive age. Screening using cytology and HPV testing is considered safe and effective during pregnancy in early detection. Colposcopy remains the gold standard in further diagnostics, with targeted biopsy indicated in selected cases. In cases of high-grade lesions (CIN II/III), conservative management is often preferred, as more than 60% of lesions regress postpartum. Invasive cervical cancer diagnosed during pregnancy is rare, with an estimated incidence of 1.4–4.6 per 100,000 pregnancies. Management decisions depend on gestational age, cancer stage, and the patient’s reproductive preference. Chemotherapy can be administered after the first trimester with acceptable maternal and fetal safety profiles. This review presents current evidence on screening, diagnostic pathways, and treatment strategies. It emphasizes the importance of individualized care, multidisciplinary collaboration, and shared decision-making to optimize outcomes for both mother and fetus.

## 1. Introduction

Pregnancy-related malignancies are defined as cancers diagnosed during pregnancy or within 12 months postpartum. Although rare, with an estimated incidence of 1 in 1000 to 1 in 2000 pregnancies, cancer during pregnancy presents a significant clinical challenge [1,2]. In recent years, the number of pregnant women diagnosed with cancer has increased, likely due to sociodemographic changes, including delayed childbearing [3]. Advanced maternal age, more common in developed countries, is a recognized risk factor for oncogenesis [4]. For example, in the United States, the mean age of first-time mothers increased from 21.4 years in 1970 to 27.5 years in 2023 [5,6].

Among pregnancy-associated cancers, the most frequent are breast cancer, cervical cancer, melanoma, ovarian tumors, and hematological malignancies [1,3]. The coexistence of pregnancy and cancer poses diagnostic and therapeutic challenges. Symptoms of malignancy are often non-specific and may be misattributed to physiological changes of pregnancy. For instance, nausea, vomiting, fatigue, anemia, constipation, and even vaginal bleeding may obscure an underlying malignancy, leading to delayed diagnosis [7,8].

Cervical cancer is the second most common malignancy diagnosed during pregnancy, with an estimated incidence of 0.1–12.0 per 10,000 pregnancies [9]. Globally, in 2022, approximately 660,000 women were diagnosed with cervical cancer, and 350,000 died from the disease [10]. Persistent infection with high-risk human papillomavirus (HPV) is the primary risk factor, with peak incidence of HPV infection occurring between ages 20 and 30, affecting up to 25% of women [11]. Cervical cancer is commonly preceded by precancerous lesions, which can be identified through cytological screening (Pap smear) [12]. Studies report spontaneous regression in 10–70% of cases postpartum, while 3–30% may progress. In approximately 25–45% of cases, the lesions persist after delivery. Management of cervical dysplasia or invasive cancer during pregnancy requires careful consideration of both maternal and fetal outcomes, often necessitating an individualized and multidisciplinary approach [11,13,14].

The aim of this narrative review is to provide a comprehensive, clinically oriented overview of available data on cervical intraepithelial neoplasia and cervical cancer diagnosed during pregnancy, with a focus on diagnostic pathways, therapeutic strategies, obstetric implications, and the latest evidence on conservative and fertility-sparing approaches.

## 2. Etiology of Cervical Dysplasia

**Figure 1:**
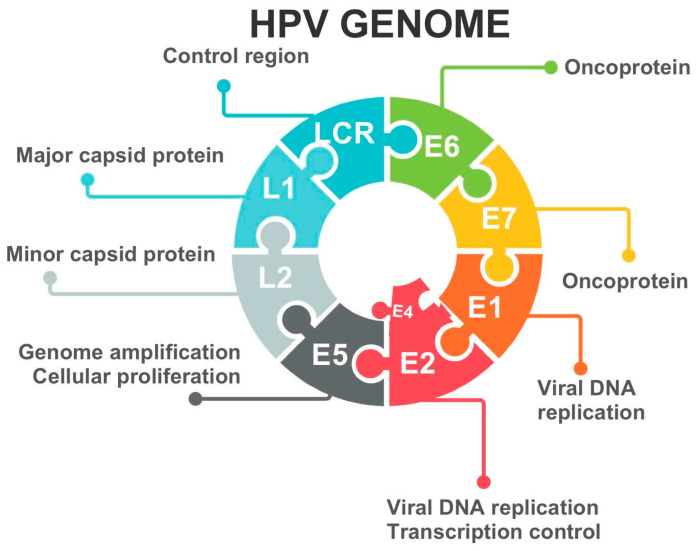
HPV (human papillomavirus) genome [15]. DNA—deoxyribonucleic acid.

The most common cause of cervical dysplasia is a long-term oncogenic infection that can generate the development of cancer cells. The virus that can cause such an infection is HPV. The most frequently mentioned are aggressive HPV types 16 and 18, and less frequently 31, 33, 35, 39, 40, 43, 51, 52, 53, 54, 55, 56, and 58, of which HPV 16 is responsible for around 50% of cervical cancer. HPV infects the basal cells of the squamous epithelium of the cervix, where viral DNA replicates as the basal cells differentiate and move to the epithelial surface. HPV gene expression becomes unrelated to the state of cellular differentiation of infected epithelial cells, and deregulation of expression of an early region of the viral genome results in a dramatic increase in expression of two HPV oncoproteins, E6 and E7 (Figure 1). The consequence is a loss of cell cycle control, and cells develop morphological features with immature squamous cells and mitotic figures in the upper half of the cervical epithelium [15,16,17,18].

**Figure 2:**
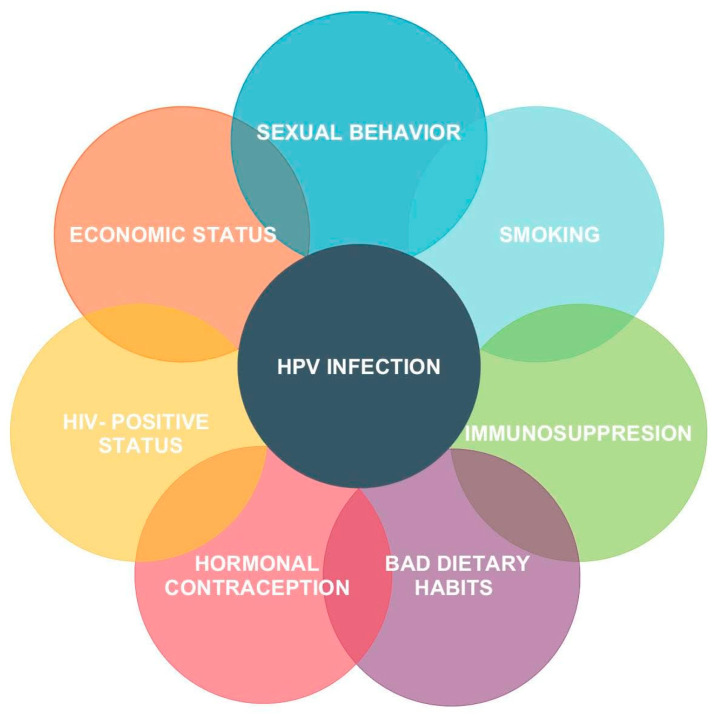
Risk factors for HPV (human papillomavirus) infection [19]. HIV—human immunodeficiency virus.

The risk factors for HPV infection are, for example, sexual behavior (casual sexual contact and low sexual culture), smoking, bad dietary habits, and immunosuppression (Figure 2). Asymptomatic carriers of HPV represent 5–20% of sexually active women of reproductive age. HPV infection is in very many cases reversible. Moreover, approximately 90% of HPV infections can resolve spontaneously within 24–36 months [19,20].

## 3. Squamous Intraepithelial Lesion in Pregnancy: Diagnostic and Therapeutic Management

According to the College of American Pathologists (CAP) and American Society of Colposcopy and Cervical Pathology (ASCCP) guidelines, the intraepithelial lesions (INs) are classified as follows: low-grade squamous intraepithelial lesion (LSIL), mild dysplasia (CIN I), high-grade squamous intraepithelial lesion (HSIL), moderate dysplasia (CIN II), and severe dysplasia (CIN III). Genital warts, which were not previously classified as CIN, were added to the LSIL group, and CIN II and CIN III were classified into one category—HSIL. In practice, CIN and SIL are often associated together and defined as LSIL (CIN I), HSIL (CIN II), or HSIL (CIN III) [21].

Moreover, cervical intraepithelial neoplasia occurs in 3.4–10% of pregnant women. In this group of patients, LSIL is most commonly found, which includes CIN I and lesions caused by HPV infection. HSIL occurs much less frequently in pregnant women, with a frequency of 0.1–1.8%. More than 60% of CIN cases in pregnant women regress spontaneously or remain stable. Due to the natural course of CIN, the only indication for treatment of CIN diagnosed during pregnancy is the suspicion of cervical cancer [22,23,24,25].

Referring to the recommendations of the American College of Obstetricians and Gynecologist (ACOG) and ASCCP, cytology should be started in women at the age of 21 and performed every 3 years. In addition, primary hrHPV testing every 5 years is also ordered in women aged 30–65 as an alternative to cytology performed every three years. If a Pap smear is scheduled as part of routine screening during pregnancy, it is generally acceptable to postpone it until after delivery—unless there are particular clinical concerns or a history of abnormal results that warrant earlier evaluation [26,27].

Grade CIN I usually regresses spontaneously if it does not intensify over about two years. It does not require any form of treatment—only constant, regular gynecological monitoring is needed. The risk of developing cancer in this population of women is very low [27]. For CIN II and CIN III, the treatment depends on the patient’s situation and the examination results, which should be discussed with the attending physician. In pregnancy, colposcopy should be performed if the estimated risk of high-grade cervical lesions (CIN III) is 4% or greater. However, immediate or expedited treatment is not advised during pregnancy. Colposcopy is appropriate in pregnant patients when the likelihood of CIN III or more severe disease exceeds 25%—a threshold where expedited treatment might typically be considered in non-pregnant patients—and especially when the risk surpasses 60%, a level at which immediate treatment would usually be preferred in those who are not pregnant. Surgical excision during pregnancy should be reserved only for situations where there is a suspicion of invasive cervical cancer. Performing a colposcopy with biopsy during pregnancy is generally safe. Clinically indicated biopsies can be performed during pregnancy. However, pregnancy causes physiological changes in the cervix that can make accurate interpretation more difficult, as these changes may resemble or mask signs of cervical cancer. This diagnostic complexity increases the risk of overlooking a malignancy. For that reason, it is preferred that colposcopic examinations during pregnancy are conducted by practitioners who have specific experience in evaluating pregnant patients [27,28].

**Figure 3:**
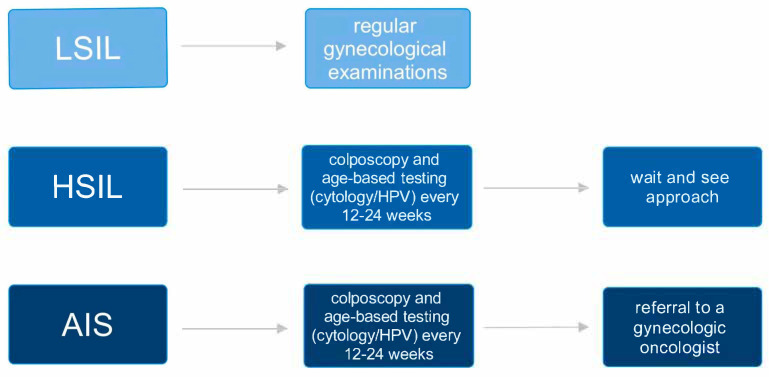
Therapeutic management depending on the grade of dysplasia [27,28]. HPV—human papillomavirus; LSIL—low-grade squamous intraepithelial lesion; HSIL—high-grade squamous intraepithelial lesion; AIS—adenocarcinoma in situ.

In cases where high-grade lesions—such as CIN II, CIN III, or adenocarcinoma in situ (AIS)—are diagnosed during pregnancy, patients should be monitored regularly through colposcopy, along with HPV testing or cervical cytology, every 12 to 24 weeks (Figure 3). The frequency of follow-up should be adapted based on factors like the stage of pregnancy, the colposcopist’s level of experience, and the patient’s risk of being lost to follow-up. If the lesion appears to worsen or shows signs suggestive of invasion, a follow-up biopsy is recommended. In the absence of concerning changes, it is acceptable to delay further colposcopic assessment until after delivery [28]. In the case of intraepithelial dysplasia-type lesions HSIL (CIN II+), a wait-and-see approach is fundamental. The rate of progression to invasive forms is very low (0–0.4%). It may be the only procedure if the examiner is convinced of the absence of invasive features. In case of doubt, a targeted biopsy is reasonable. Cervical scraping or ablation is not recommended. Conization should be performed if there are inconsistencies between cytology and colposcopic examination raising suspicion of invasion. In cases where AIS is identified, referral to a gynecologic oncologist is the preferred course of action. However, it is also acceptable for a gynecologist with advanced expertise in colposcopic evaluation and management of AIS to oversee care [11,29,30].

Appropriate treatment is deferred until the post ancestral period, especially as these lesions often undergo spontaneous regression (48–70% of cases) [11,29,30]. In addition, there are no oncological indications for Caesarean section. Postpartum colposcopy should be scheduled at least four weeks after delivery to allow adequate cervical healing and to ensure the procedure can be completed within the timeframe of postpartum insurance coverage. If a lesion is identified during the postpartum colposcopy, management should include either an excisional procedure or a comprehensive diagnostic workup—consisting of cytology, HPV testing, colposcopic assessment, and biopsy. In cases where no visible lesions are present, a full diagnostic evaluation is still recommended to rule out underlying disease [27]. Besides, the studies reveal that the cervical dysplasia during pregnancy was not associated with pregnancy complications [12,31]. In pregnant women, diagnostic procedures are carried out to exclude cervical cancer. For precancerous lesions such as HSIL (CIN II+), it is recommended to postpone definitive treatment until after childbirth [11,32,33].

## 4. Cervical Cancer

Cervical cancer is the fourth most common cancer in women, with an estimated 600,000 new cases in 2020 [34]. The most prevalent histological subtypes are squamous cell carcinoma and adenocarcinoma, accounting for about 70% and 25% of all cases, respectively [35]. The main etiological factor responsible for the development of cervical cancer is infection with oncogenic subtypes of HPV-16 and -18. The infection is sexually transmitted; therefore, the main risk factors include a large number of sexual partners and early onset of sexual life. In addition, age under 18 years at the time of pregnancy and multiple pregnancies are indicated as risk factors for HPV infection and cervical cancer. Smoking also contributes to the increased incidence of cervical cancer [36].

Cervical cancer is the most common gynecologic cancer diagnosed during pregnancy, with an estimated incidence of 1.4–4.6 cases per 100,000 pregnancies [37]. The clinical presentation of cervical cancer in pregnant women depends on the clinical stage and size of the tumor. In the early stages, most cases are asymptomatic or sparsely symptomatic. The most prominent symptoms are irregular or heavy vaginal bleeding, especially after sexual intercourse. In some patients, the first symptom is abnormal vaginal discharge, which could be watery, mucous, or purulent and malodorous. In late stages, pelvic pain or chronic anemia due to prolonged irregular vaginal bleeding predominates. These symptoms can make diagnosis difficult, as they also occur in the course of other diseases during pregnancy [36,38].

Diagnosis of cervical cancer in a pregnant woman includes cytology, colposcopy, and cervical biopsy. Histopathological examination is required to confirm the diagnosis. The guidelines recommend colposcopic biopsy without cervical curettage. It is estimated that the risk of HGSIL progression to invasive cancer during pregnancy is less than 2% [37]. Nevertheless, the effect of pregnancy on cervical cancer progression remains controversial. Increased levels of estrogen, progesterone, and human chorionic gonadotropin, as well as local immunosuppression during pregnancy, may induce HPV reactivation, indirectly suggesting that pregnancy may promote cervical cancer progression. Additionally, increased uterine blood circulation and cervical dilatation during childbirth may increase the risk of spreading cancer cells and accelerate cervical cancer progression [39]. Moreover, most cases of cervical cancer in pregnancy are stage I detected, allowing the pregnancy to continue and providing a greater chance of achieving complete remission [37]. Recent evidence suggests that pregnancy is not an adverse prognostic factor for mothers and newborns. Studies have shown no difference in oncologic prognosis for women with cervical cancer diagnosed during pregnancy compared to women who are not pregnant [39,40]. Moreover, children born to women with invasive cervical cancer were similar in terms of gestational age and prematurity rates [41].

The diagnosis of invasive cervical cancer in pregnancy requires multidisciplinary management. The aim of treatment is both oncologic and perinatal care, as well as fetal survival, without risk of morbidity [37].

## 5. Management of Cervical Cancer During Pregnancy

The treatment of cervical cancer in non-pregnant women primarily depends on the stage of cancer according to the International Federation of Gynecology and Obstetrics (FIGO) classification (Table 1). Staging is based on tumor size, vaginal or parametrial involvement, extension to the bladder or rectum, and the presence of distant metastases. In pregnant patients, additional considerations include gestational age (determined by ultrasonography) and the patient’s decision regarding pregnancy continuation [29]. Diagnostic procedures in pregnancy should adhere to standard oncologic principles, including FIGO staging, colposcopic evaluation, assessment of lymph node involvement, and imaging—preferably magnetic resonance imaging (MRI) [38,42]. In contrast to non-pregnant patients, staging modalities involving ionizing radiation—such as computed tomography (CT) or positron emission tomography (PET-CT)—are contraindicated during pregnancy due to their teratogenic potential. Likewise, gadolinium-based contrast agents used in MRI are generally avoided because of associations with stillbirth and immune-related conditions [43]. Nevertheless, the 11th European Symposium on Urogenital Radiology concluded that gadolinium administration during pregnancy may be justified when the expected clinical benefit outweighs potential risks [44]. Lymph node assessment is optimally performed by laparoscopic pelvic lymphadenectomy before 22 weeks of gestation, as lymph node status is a crucial prognostic factor guiding treatment planning. While CT is typically used for lymph node evaluation in non-pregnant women, it is not suitable during pregnancy, and MRI offers limited sensitivity. Therefore, laparoscopic assessment remains the preferred method [43,45].

A key factor influencing treatment decisions is the patient’s desire to continue the pregnancy. If the patient chooses not to continue the pregnancy, standard treatment according to protocols for non-pregnant women should follow. In women who do not wish to continue the current pregnancy but hope for future fertility, fertility-sparing treatment may be considered up to stage IIA1, provided the tumor is <2 cm in diameter. For more advanced stages, such approaches are strongly discouraged [29].

**Figure 4:**
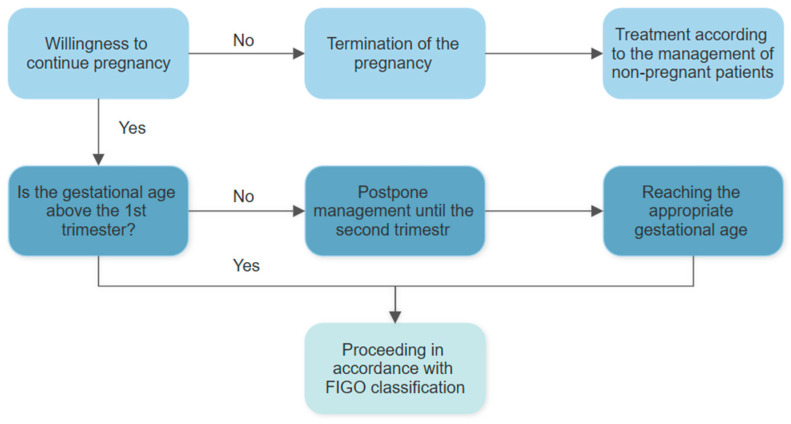
Management depending on the patient’s desire to preserve pregnancy [29]. FIGO—International Federation of Gynecology and Obstetrics.

When selecting a treatment approach, maintaining the longest possible intrauterine development of the fetus is also a priority [29,47]. Management strategies vary depending on the gestational age at the time of diagnosis (Figure 4). During the first trimester, it is generally recommended to postpone treatment until the second trimester. The second trimester is typically considered the safest time for surgical procedures, such as laparoscopic surgery. At this stage, the fetus is still small enough to allow surgical access to the abdominal area, and the likelihood of miscarriage or preterm labor is lower than in the first or third trimesters [29].

Recent evidence supports the growing role of fertility-preserving strategies in the management of early-stage cervical cancer. For tumors smaller than 2 cm, without lymphovascular space invasion, deep stromal infiltration, or nodal involvement, conservative approaches—such as cervical conization or simple trachelectomy—combined with sentinel lymph node mapping offer effective oncologic control while preserving fertility potential [48]. These strategies are increasingly preferred, particularly following the results of the Simple Hysterectomy and Pelvic Node Assessment (SHAPE) trial, which demonstrated that less radical surgery can provide comparable oncologic outcomes with reduced morbidity [49]. This reflects a broader shift toward personalized care—especially during pregnancy—where gestational age, maternal preferences, and fetal safety must be carefully balanced [48].

For FIGO stage IA1, cervical conization is recommended between 22 and 25 weeks of gestation [22,38]. A retrospective study demonstrated that patients who underwent cervical conization with a depth exceeding 1 cm exhibited a higher incidence of preterm birth and low-birth-weight infants compared to those without a history of conization. Prophylactic cervical cerclage may be considered in these cases to mitigate the risk of preterm delivery and reduce intraoperative bleeding [38]. In case of positive margins, it is recommended to repeat the conization procedure [29]. For stages IA2 to IB1, characterized by a tumor size of up to 2 cm, pelvic lymphadenectomy is a key component of surgical staging and treatment planning. When performed laparoscopically, this procedure enables accurate assessment of lymph node involvement while minimizing surgical morbidity [29]. Where there is no lymph node involvement, it is advised to delay conization or perform a simple trachelectomy after reaching fetal maturity. Radical trachelectomy or other extensive cervical resections during pregnancy are not advised due to the high risk of miscarriage.

In support of individualized care, Ferrari et al. retrospectively compared cold knife (CK) conization and carbon dioxide (CO_2_) laser conization in 1270 women treated for preinvasive cervical lesions [50]. CO_2_ laser conization was associated with a significantly lower rate of positive endocervical or deep margins (4.3% vs. 13.3% with CK conization), indicating enhanced surgical precision. However, in cases of incidental cervical cancer, CK conization resulted in fewer positive margins (22.7% vs. 58.8%), suggesting its suitability in patients with suspected AIS. Both methods demonstrated comparable oncologic outcomes among women pursuing fertility preservation. These findings underscore the importance of individualized, risk-adapted strategies in the conservative treatment of early-stage cervical cancer—balancing oncologic safety with reproductive goals and long-term quality of life [50].

**Figure 5:**
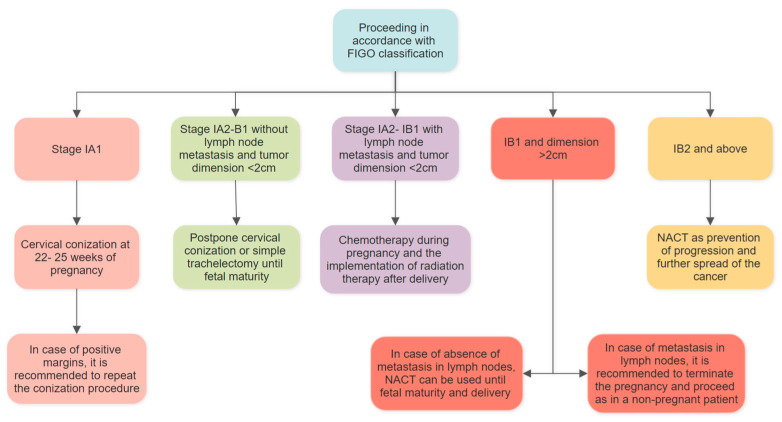
Proceeding in accordance with FIGO classification [38]. FIGO—International Federation of Gynecology and Obstetrics; NACT—neoadjuvant chemotherapy.

If the lymph nodes are involved and the dimension of the tumor is less than 2 cm, the European Society of Clinical Oncology recommends the use of chemotherapy during pregnancy and the implementation of radiation therapy after delivery. In IB1 and dimension greater than 2 cm, removal of pelvic and abdominal lymph nodes is indicated. In the absence of their involvement, it is believed that neoadjuvant chemotherapy (NACT) can be used until fetal maturity and delivery. When, however, nodes are positive, it is recommended to terminate the pregnancy and proceed as in a non-pregnant patient. In stage IB2 and above, NACT is thought to be a method to prevent progression and further spread of the cancer (Figure 5) [38,51].

The NACT regimen, which is usually used, is the combination of cisplatin and paclitaxel because both have the lowest risk of adverse effects. Moreover, carboplatin is recommended to use instead of cisplatin to avoid children’s ototoxicity. Administration of taxanes during pregnancy is probably safe; the most common adverse effects are anaphylactic reactions or preterm complications. Besides taking chemotherapeutics, the ondansetron and corticosteroids are administered as premedication [52]. According to European Society of Gynaecological Oncology (ESGO) guidelines, the most common chemotherapy schedules include cisplatin monotherapy or combinations with vincristine, paclitaxel, adriamycin, etoposide, and bleomycin. Available combinations are vincristine + bleomycin or etoposide + dactinomycin + cyclophosphamide [53]. The dosage and chemotherapy scheme are the same as for non-pregnant women and are based on actual weight and height. It is important to weigh, physically examine, and perform obstetrical ultrasonography at every new cycle of chemotherapy [43]. Cisplatin is dosed at 50 mg/m^2^ intravenously (IV) every 3 weeks in monotherapy. It is worth emphasizing that patients must be well-hydrated before cisplatin administration. In combination with cisplatin, the recommended dosage of paclitaxel is 135 mg/m^2^ over 24 h on day 1, followed by cisplatin 50 mg/m^2^ IV infusion over 60 min. In combination with carboplatin, the recommended dosage of paclitaxel is 175 mg/m^2^ over 3 h on day 1, immediately followed by carboplatin infusion at an AUC of 5 mg/mL/min over 1 h on the same day [54].

Chemotherapy in cervical cancer during pregnancy is aimed at stabilizing the disease, reducing the size of the tumor, increasing its radiosensitivity, and preventing lymph node metastasis during pregnancy until delivery. All of these chemotherapy measures are aimed at the patient’s ability to carry a pregnancy while controlling the disease [55,56].

The effect of chemotherapy on the fetus is a major concern for attending physicians. It is known that the period of greatest fetal sensitivity to cytostatic is during organogenesis, which lasts from the 6th to 10th week of pregnancy. For this reason, it is not recommended to administer chemotherapeutics to a patient before 10 weeks of pregnancy. Chemotherapy in the second and third trimesters is associated with intrauterine fetal growth abnormalities, low birth weight, and preterm delivery [57]. After the period of organogenesis is over, chemotherapy can still affect the central nervous system, the hematopoietic system of the gonads, or the eyes [38,55]. It is worth emphasizing that after chemotherapy, it is important to measure the peak systolic velocimetry of the middle cerebral artery because it is considered a very accurate method of fetal anemia screening [52,58].

Due to the effects of chemotherapy on the bone marrow, delivery should be scheduled in advance for a date no earlier than 3 weeks before the last cycle of chemotherapy. This is a duration that will allow for bone marrow renewal, whose proper hematopoietic function in both mother and child is important in case of intrauterine hemorrhages or infections [55].

The management of cervical cancer during pregnancy involves analyzing fetal well-being and the risks associated with chemotherapy. Therefore, a delaying strategy may be considered, especially when the tumor is diagnosed before 22 gestational weeks and is in the IA2-IB3 stage. Nevertheless, the European Society For Medical Oncology (ESMO) guidelines recommend delaying treatment in all early stages. In contrast, in cases of progression, appropriate therapy must be advocated [52]. According to the ESGO guidelines, a review of 100 cases of delayed treatment, ranging from 3 to 56 weeks, in FIGO stage I–II, showed that only 4 patients died from the disease. Additionally, another analysis of studies involving 70 patients with FIGO stage I–II who delayed treatment for more than 6 weeks found that only 2 patients died from the disease. Sixteen patients were diagnosed in the first trimester [53].

## 6. Conclusions

Cervical dysplasia is mainly associated with long-term infection with oncogenic HPV types 16 and 18. Approximately 90% of HPV infections can resolve spontaneously within 24–36 months. More than 60% of CIN cases in pregnant women resolve spontaneously or remain stable. In addition, studies show that cervical dysplasia during pregnancy is not associated with pregnancy complications. For HSIL, treatment decisions depend on the individual patient’s circumstances and test results, which should be discussed with the attending physician. In the management of cervical cancer, treatment in non-pregnant women is primarily determined by the FIGO staging system, which considers tumor size, local invasion, and metastasis. However, in pregnant women, additional considerations such as gestational age and the patient’s willingness to continue the pregnancy play a crucial role in planning care. When accurate lymph node staging is required, laparoscopic biopsy before 22 weeks of gestation is recommended. Fertility-sparing strategies, such as conization or simple trachelectomy, may be applied in highly selected early-stage cases, ensuring careful staging, multidisciplinary oversight, and individualized care. Surgical procedures are typically safest during the second trimester. In the case of pregnant patients with cervical cancer, chemotherapy can be used to stabilize the disease until the pregnancy ends. The scheduling and dosage of chemotherapy during pregnancy are generally similar to those for non-pregnant women. In many cases, systemic treatment involves cisplatin either as monotherapy or in combination with taxanes. Treatment may be delayed in some cases, particularly in early-stage carcinoma, but patients must be closely monitored for tumor progression. Given the complexity of diagnosis and treatment, care should be centralized in tertiary referral centers with access to gynecologic oncologists, colposcopists, and maternal-fetal medicine specialists.

## Figures and Tables

**Table 1:** FIGO (International Federation of Gynecology and Obstetrics) staging of cervical cancer [46].

Stage	Description
I	Carcinoma confined to the cervix
IA	Diagnosed only by microscopy, and the maximal invasional depth ≤ 5 mm
IA1	Measured stromal invasion ≤ 3 mm in depth
IA2	Measured stromal invasion > 3 mm and ≤5 mm in depth
IB	The deepest invasion measurement of >5 mm (deeper than stage IA) only cervical lesion
IB1	Clinically visible lesion >5 mm deep and ≤2 cm in greatest dimension
IB2	Clinically visible lesion > 2 and ≤4 cm in greatest dimension
IB3	Lesion > 4 cm in greatest dimension
II	Carcinoma invasion beyond the uterus, without the lower 1/3 part of the vagina or pelvic wall
IIA	The tumor involves only the upper 2/3 of the vagina
IIA1	The tumor involves only the upper 2/3 of the vagina and is ≤4 cm in greatest dimension
IIA2	The tumor involves only the upper 2/3 of the vagina and is >4 cm in greatest dimension
IIB	With parametrial invasion
III	Carcinoma extends to the pelvic wall and/or involves the lower third of the vagina and/or causes hydronephrosis or renal failure and/or involves pelvic and/or para-aortic lymph nodes
IIIA	The tumor involves a third of the vagina without reaching the pelvic wall
IIIB	The tumor involves the pelvic wall and/or causes hydronephrosis or renal failure
IIIC	Pelvic and/or para-aortic lymph node involvement
IIIC1	Pelvic lymph node metastasis only
IIIC2	Para-aortic lymph node metastasis
IV	The tumor has extended beyond the true pelvis or has involved bladder or rectal mucosa
IVA	The tumor spread to adjacent pelvic organs
IVB	The tumor spread to distant organs

## Data Availability

The data used in this article are sourced from materials mentioned in the References section.

## Abbreviations

The following abbreviations are used in this manuscript:

HPV	human papillomavirus
CIN	cervical interepithelial neoplasia
LSIL	low-grade squamous intraepithelial lesion
HSIL	high-grade squamous intraepithelial lesion
AIS	adenocarcinoma in situ
MRI	magnetic resonance imaging
CT	computed tomography
PET	position-emitting tomography
CK	cold knife
CO_2_	carbon dioxide
NACT	neoadjuvant chemotherapy
